# Coding-Complete Genome Sequence of Murine Hepatitis Virus Strain 3 from Brazil

**DOI:** 10.1128/MRA.00248-21

**Published:** 2021-04-15

**Authors:** Amanda Barbosa Garcia, Ana Paula de Moraes, Daniele Masselli Rodrigues, Rovilson Gilioli, Edmilson Ferreira de Oliveira-Filho, Ricardo Durães-Carvalho, Clarice Weis Arns

**Affiliations:** a Laboratory of Virology, University of Campinas (UNICAMP), Campinas, São Paulo, Brazil; b Laboratory of Animal Quality Control, CEMIB, University of Campinas (UNICAMP), Campinas, São Paulo, Brazil; c Charité-Universitätsmedizin Berlin, Corporate Member of Freie Universität Berlin, Humboldt-Universität zu Berlin, and Berlin Institute of Health, Institute of Virology, Berlin, Germany; KU Leuven

## Abstract

Murine hepatitis virus (MHV) strain 3, one of the most important inducers of viral hepatitis, has been extensively studied as an organism to gain a better understanding of coronavirus biology and pathogenesis. Only one sequence is currently available. Another representative isolate has now been sequenced and added to the arsenal of MHV-3 variants.

## Abstract (precis)

Murine hepatitis virus (MHV) strain 3, one of the most important inducers of viral hepatitis, has been extensively studied as an organism to gain a better understanding of coronavirus biology and pathogenesis. Only one sequence is currently available.

## ANNOUNCEMENT

The group of coronaviruses (CoV) includes several species that infect a variety of hosts (1). Murine hepatitis virus (MHV) belongs to the family *Coronaviridae*, subfamily *Orthocoronavirinae*, and genus *Betacoronavirus* and contains a positive-sense single-stranded RNA genome sequence approximately 25 to 31 kb in size (2). With Mus musculus as the main host, MHV encompasses a set of well-described more virulent (MHV-2, MHV-3, MHV-A59, and MHV-JHM) and less virulent (MHV-1, MHV-S, MHV-Y, and MHV-Nu) strains (3). It is noteworthy that, since MHV shares the same genus (β-coronavirus) as severe acute respiratory syndrome coronavirus 2 (SARS-CoV-2), the causative agent of coronavirus disease 2019 (COVID-19), MHV together with mouse models could offer, through a translational approach, mechanistic insight into the SARS-CoV-2 biology and pathogenesis (3, 4).

MHV-3, stored at the Laboratory of Virology, University of Campinas (UNICAMP), Brazil, originally from the Culture Collection Laboratory of Animal Quality Control, Multidisciplinary Center for Biological Investigation on Laboratory Animal Science (CEMIB), UNICAMP, was grown on cell line L-929 (ATCC CCL-1). RNA was extracted from the supernatant (centrifugation at 2,000 rpm for 10 min) using a viral RNA minikit (Qiagen), and cDNA synthesis was performed using random primers with the high-capacity cDNA reverse transcription kit (Applied Biosystems), according to the manufacturer’s instructions. PCR was performed with primers designed from the primalscheme tool (ZiBRA project), aiming to produce 36 overlapping amplicons (Table 1), and their products were purified and quantified using the ExoSAP-IT Express PCR product cleanup reagent and Qubit v.2.0 fluorometer (Invitrogen), respectively. Next, the MHV-3 coding genome was Sanger sequenced at both ends using a primer walking approach. Genome assembly and full annotation (based on GenBank accession numbers FJ647224.1 and NC_048217.1) were performed using the Unipro UGENE v.37.1, Geneious v.9.1, and ORFfinder (https://www.ncbi.nlm.nih.gov/orffinder/) platforms.

**TABLE 1:** Primers used for whole-genome sequencing of MHV-3 from UNICAMP

Primer	Sequence	Size (nucleotides)	Primer position^*a*^
MHV-3_1_LEFT	5′-ACTTTATAAACGGCACTTCCTGC-3′	23	47–1048
MHV-3_1_RIGHT	5′-GCCACACAGGGAATAACTCCTT-3′	22	
MHV-3_2_LEFT	5′-GCTATCGCGGTGTTAATCCCAT-3′	22	917–1935
MHV-3_2_RIGHT	5′-GCGGGGCACTAAACCATCTAAT-3′	22	
MHV-3_3_LEFT	5′-AATAGGGGCGACTACAGTCTCC-3′	22	1804–2808
MHV-3_3_RIGHT	5′-AAACTCTGAACACGCCTTCGAA-3′	22	
MHV-3_4_LEFT	5′-TCAATGCTGGAGGTTTCCTTGC-3′	22	2658–3694
MHV-3_4_RIGHT	5′-TAATTGACTTGGGCACGCTCTT-3′	22	
MHV-3_5_LEFT	5′-GCTGGCTGCGTTCTACTTTGAT-3′	22	3548–4568
MHV-3_5_RIGHT	5′-ACATTCTTTGTCACAACACCAAGT-3′	24	
MHV-3_6_LEFT	5′-ACTTTTAGAACGTGCCTATCAGCA-3′	24	4437–5447
MHV-3_6_RIGHT	5′-GACACAAACCTTAGTGGCTTGC-3′	22	
MHV-3_7_LEFT	5′-AAGCAGGCGAATAACAATTGCT-3′	22	5314–6305
MHV-3_7_RIGHT	5′-CTCAAAGATGCATCACCATGGC-3′	22	
MHV-3_8_LEFT	5′-TTACAGAATGGCCAGCAGCTAC-3′	22	6173–7198
MHV-3_8_RIGHT	5′-TTCTATAGCCCACATCCGATGC-3′	22	
MHV-3_9_LEFT	5′-TCTGCCTAATATTGGATTCTTCCCT-3′	25	7080–8087
MHV-3_9_RIGHT	5′-TCCACCATCAGCATCGGTCTAT-3′	22	
MHV-3_10_LEFT	5′-GTGTGCCTGAAACCCATGTAGT-3′	22	7976–8944
MHV-3_10_RIGHT	5′-TAACAGCAACCACAACAGGACA-3′	22	
MHV-3_11_LEFT	5′-TAATGGTGTGCTACGGGATGTG-3′	22	8805–9825
MHV-3_11_RIGHT	5′-GTTTGAAACAACGACTGCTGCA-3′	22	
MHV-3_12_LEFT	5′-CAACCCTTTATTTCCCATCGGAGA-3′	24	9707–10672
MHV-3_12_RIGHT	5′-CGGTGTGACAACCAGTACTCAA-3′	22	
MHV-3_13_LEFT	5′-CCCAAGGAGCCTTCCATGTTAC-3′	22	10517–11521
MHV-3_13_RIGHT	5′-AGTACCACATAGACACCAGGCT-3′	22	
MHV-3_14_LEFT	5′-GGTGTCGTGTTGCTAGTTGCTA-3′	22	11407–12377
MHV-3_14_RIGHT	5′-ATACGTTCGAGCTTACGTGCAA-3′	22	
MHV-3_15_LEFT	5′-AGGCTAGTGGCTCTGCTAATCA-3′	22	12266–13232
MHV-3_15_RIGHT	5′-CAATTAGTGACAGGAGCGCCAC-3′	22	
MHV-3_16_LEFT	5′-ATCGTCGACGGTAAGATTGCAG-3′	22	13089–14088
MHV-3_16_RIGHT	5′-CAGTGTTAAGCAGGGCCCTATT-3′	22	
MHV-3_17_LEFT	5′-CCTATGCTGAGTGTGAAGAGTCC-3′	23	13963–14969
MHV-3_17_RIGHT	5′-ACACCCACCCTCATAGATCTCA-3′	22	
MHV-3_18_LEFT	5′-AGGATGGTAATGCTGCTATTACTGA-3′	25	14839–15828
MHV-3_18_RIGHT	5′-CCTTGGACGCGAACTCTGAATT-3′	22	
MHV-3_19_LEFT	5′-TGACCCCGCATTTGTTAGTGAG-3′	22	15719–16701
MHV-3_19_RIGHT	5′-ACAATTTAAGGCGCTCGGTACA-3′	22	
MHV-3_20_LEFT	5′-ACAATCTTGTACTGGTTCGCCC-3′	22	16586–17548
MHV-3_20_RIGHT	5′-GTTCCTTTATTCAGCAGCACACG-3′	23	
MHV-3_21_LEFT	5′-CCCGAGTTGGTGACTGACATTA-3′	22	17391–18407
MHV-3_21_RIGHT	5′-CCCAATGCTATCACGTATCGCA-3′	22	
MHV-3_22_LEFT	5′-GCTTGACTTGACCCTTGATGGT-3′	22	18284–19295
MHV-3_22_RIGHT	5′-CGTGTCGAACCTACACACAACT-3′	22	
MHV-3_23_LEFT	5′-ATGATGCCTCGCCTGTTGTTAA-3′	22	19147–20149
MHV-3_23_RIGHT	5′-ACAACTACGCCATTTAGCTCGG-3′	22	
MHV-3_24_LEFT	5′-TGATGGTCGTGATAATGGTGCT-3′	22	20018–21018
MHV-3_24_RIGHT	5′-CCTTATCTGACCCTGCACCAAG-3′	22	
MHV-3_25_LEFT	5′-CTCTGGAATTATGGCAAGCCGA-3′	22	20868–21902
MHV-3_25_RIGHT	5′-TCCACCTGTTTGTATTGTTCTGCT-3′	24	
MHV-3_26_LEFT	5′-TCCATTGGCCCAATTTAGTGGC-3′	22	21753–22775
MHV-3_26_RIGHT	5′-ACCTGAATGGCACAACTCTTGG-3′	22	
MHV-3_27_LEFT	5′-TGATGACTGGTTCCTCTTTGGC-3′	22	22664–23636
MHV-3_27_RIGHT	5′-CGTGCAAGGAAATACACTGCAC-3′	22	
MHV-3_28_LEFT	5′-TGTCAGCCGCCATATTGTTTCT-3′	22	23519–24528
MHV-3_28_RIGHT	5′-ACGCATAAAAAGTACCACCCTGT-3′	23	
MHV-3_29_LEFT	5′-GCTTTTGGCACACAGATGTCAA-3′	22	24409–25420
MHV-3_29_RIGHT	5′-CTCCTAGAACATTCCCGATGCG-3′	22	
MHV-3_30_LEFT	5′-TGCCTATGCCCAGCAATGTTTT-3′	22	25277–26275
MHV-3_30_RIGHT	5′-CCATCAATGGCTTGAACACTATCA-3′	24	
MHV-3_31_LEFT	5′-GGTGCTGGACTATGCGTTGATT-3′	22	26145–27119
MHV-3_31_RIGHT	5′-AGAAGCACTAATAGCGCCAAAC-3′	22	
MHV-3_32_LEFT	5′-TGGGTTCGATGCAACCAATTCT-3′	22	26999–27977
MHV-3_32_RIGHT	5′-ATCCTGGTGTCCTCCATACTCA-3′	22	
MHV-3_33_LEFT	5′-GGCCTTGGTACATTTGGTTGCT-3′	22	27832–28823
MHV-3_33_RIGHT	5′-TTTCGAGCAACAAGGCCCTAAA-3′	22	
MHV-3_34_LEFT	5′-CGAAAAATCAGGCCACCCAAAA-3′	22	28696–29689
MHV-3_34_RIGHT	5′-AAAACCGCTAACACCGTCTACC-3′	22	
MHV-3_35_LEFT	5′-GTGGCCACCTCTATATGCAAGG-3′	22	29544–30579
MHV-3_35_RIGHT	5′-GACTTCTTTGGCGCTTTGCTTT-3′	22	
MHV-3_36_LEFT	5′-CGTGGGCCAAATAATCGCTCTA-3′	22	30418–31400
MHV-3_36_RIGHT	5′-GGGCATTGCAGGAATAGTACCC-3′	22	

a Based on the MHV-3 sequence found under GenBank accession number FJ647224.1.

**FIG 1:**
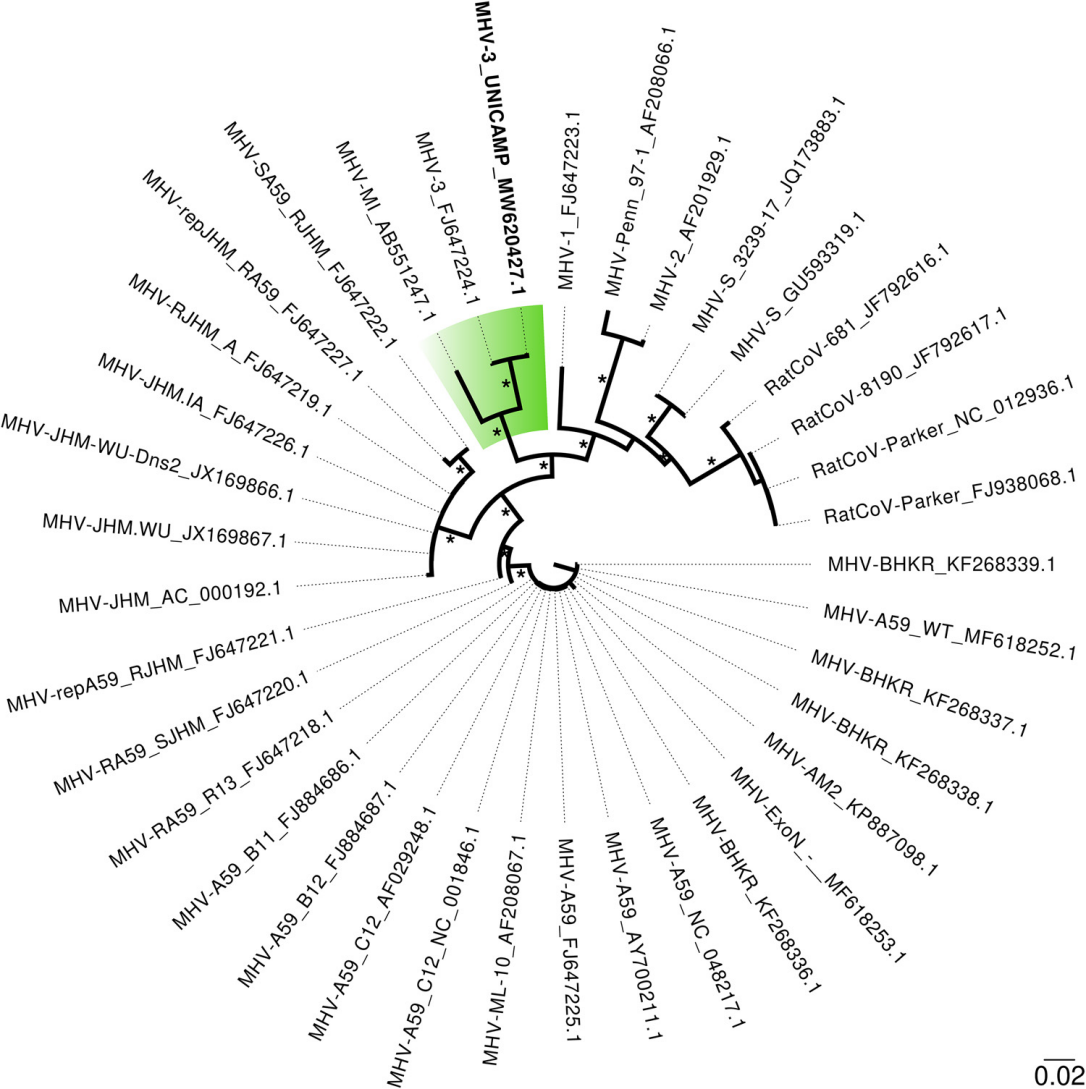
Maximum likelihood phylogenetic tree based on the 36 representative genome sequences of murine coronavirus strains. MHV-3, the strain from this study, is highlighted and in bold. The labels include GenBank accession numbers. The asterisks indicate 100% of SH-aLTR/aBayes/ultrafast bootstrap support values. The branch lengths are drawn to scale of nucleotide substitutions per site according to the bar in the figure.

The final consensus of the MHV-3 genome sequence (length, 31,218 nucleotides; GC content, 41.97%) obtained from UNICAMP shares 99.81% nucleotide identity with the only available MHV-3 full-genome sequence, from the J. Craig Venter Institute in Rockville, MD (GenBank accession number FJ647224.1). All murine CoV whole-genome sequences (*n =* 36) were retrieved from the GenBank database, aligned using MAFFT v.7 (Web version), and manually edited (Unipro UGENE v.37.1). Phylogenetic analysis using the maximum likelihood method (IQ-TREE) indicated that it groups (100% of SH-aLRT/aBayes/ultrafast bootstrap support values) (5) with MHV-MI (AB551247.1), a virus closely related to the highly virulent strain MHV-JHM (6) (Fig. 1). All tools were run using default parameters unless otherwise specified. Lastly, with this MHV-3 strain from UNICAMP sequenced, the scientific community now has a new standard for comparison for further studies involving murine CoV-induced hepatitis.

### Data availability.

The sequence of MHV-3 has been deposited in the GenBank database under accession number MW620427.
